# Preparation of novel and recyclable chitosan-alumina nanocomposite as superabsorbent to remove diazinon and tetracycline contaminants from aqueous solution

**DOI:** 10.1016/j.heliyon.2023.e23139

**Published:** 2023-12-09

**Authors:** Amir Adibzadeh, Mohammad Reza Khodabakhshi, Ali Maleki

**Affiliations:** a Health Research Center, Life Style Institute, Baqiyatallah University of Medical Sciences, Tehran, Iran; b Applied Biotechnology Research Center, Baqiyatallah University of Medical Sciences, Tehran, Iran; c Catalysts and Organic Synthesis Research Laboratory, Department of Chemistry, Iran University of Science and Technology, Tehran, Iran

**Keywords:** Efficient adsorbent, Chitosan, Boehmite, Diazinon, Tetracycline, Reusability

## Abstract

This work presents a novel, strong and efficient adsorbent (CS@TDI@EDTA@γ-AlO(OH)) prepared through the green process using three components, chitosan, BNPs and EDTA using amide and ester bridges. An eco-friendly and easy approach was used for the preparation of this novel adsorbent, the low cost, easy access to the used materials, and the simplicity of the preparation method are some of the interesting advantages of this work. Also, this prepared adsorbent was used as an adsorbent to remove diazinon organophosphate poison and tetracycline antibiotic from aqueous solutions. In order to confirm the prepared adsorbent structure, the CS@TDI@EDTA@γ-AlO(OH) composite was investigated by various analyses including FT-IR, EDX, XRD, FESEM and TGA. The adsorption behavior of the adsorbent prepared for the removal of tetracycline and diazinon was investigated under different conditions by varying the concentration, temperature, the adsorbent dose, pH and contact time. Based on various tests, the highest diazinon adsorption capacity was obtained for 0.12 g/L adsorbent at pH 7 and 60 °C with 40 mg/L initial concentration. Also, the maximum adsorption capacity of the tetracycline was obtained for 0.12 g/L adsorbent at pH 9 and 60 °C with 30 mg/L initial concentration. The equilibrium results for diazinon and for tetracycline were in good accordance with the Langmuir and Freundlich isotherm models, respectively. Also, the highest adsorption capacities for diazinon at pH 7 and tetracycline at pH 9 were 1428.5 and 555.5 mg/g, respectively. Also the kinetic investigations revealed that the correlation factor (R^2^) of pseudo-second-order model obtained for the adsorption of diazinon and tetracycline was 0.9986 and 0.9988, while the coefficient k (g/mg.min) was 0.000084 and 0.0033, respectively. These results indicate that the adsorption of diazinon and tetracycline is pseudo-second-order kinetics model. Formation of hydrogen bonds between adsorbate and adsorbent as well as the high specific surface area and porosity of the adsorbent are the main mechanisms that contribute to the adsorption process. In addition, thermodynamic studies indicated that the adsorption of diazinon and tetracycline is a spontaneous endothermic process. The adsorbent prepared in this work was expected to have wide range of applications in wastewater treatment thanks to its good reusability in water and strong removal of diazinon and tetracycline compared to other adsorbents.

## 1 Introduction

Due to the incomplete removal of poisons and medicinal substances from wastewater in the wastewater treatment plants, they are discharged into rivers through sewage and dewatering sludge [[1], [2], [3], [4], [5], [6], [7], [8], [9], [10], [11], [12], [13], [14], [15], [16], [17]]. As a result, they remain in the environment, water resources, and food resources as pollutants. Also, wastewater treatment only purifies a part of the poisons and pharmaceutical compounds in the water, and a large amount of them remain in the underground and surface water [[18], [19], [20], [21], [22], [23], [24], [25], [26], [27]].

To remove pollutants in water from various methods such as electro-deposition [28,29], membrane filtration [[30], [31], [32]], biological process [33,34], ion exchange [35,36], photo catalysis [[37], [38], [39], [40]], oxidation [41,42] and adsorption [[43], [44], [45]] are used, which among the mentioned methods, surface adsorption is known as the most reliable method, the simplest and most efficient method to remove contaminants.

In recent years, nano-adsorbents has received much attention for removing pollutants from aqueous solutions [46,47]. Nano-adsorbents show high adsorption capacity due to their high surface area and the presence of a great number of active sites.

Among medicinal contaminants, antibiotics are commonly found in water due to their wide use in the medical field. Tetracycline (TC) is one of the medicinal antibiotics which is used for treating many bacterial diseases in animals and humans [[48], [49], [50], [51], [52], [53]]. In domestic wastewater, the concentration of antibiotics is usually between ng/L to μg/L, and in the sewage effluents of farms, hospitals and pharmaceutical industries, this amount has been up to 500 mg/L [[54], [55], [56], [57], [58]]. The antibiotic resistance genetically occurs as a result of mutations or the acquisition of new resistance genes via horizontal gene transfer mechanisms. The antibiotic resistance is mainly related to the following reasons: (i) inadequate regulations and imprecise usage, (ii) unnecessary use of antibiotics, (iii) poor hygiene/sanitation, (iv) manure/feces which contain nonmetabolized antibiotics, and (v) misuse of antibiotics to promote growth in livestock and poultry [[59], [60], [61]]. Incorrect and excessive use of antibiotics in veterinary, agriculture, and medical sectors can enhance the global antimicrobial resistance which increases the concern about the role of the environment as an antimicrobial resistance reservoir in the propagation of antimicrobial resistance genes [62,63]. Consequently, the prevention of antibiotics release into the environment is of high importance.

Organophosphorus poisons are an important class of pesticides to control all kinds of harmful insects in agriculture [[64], [65], [66], [67], [68]]. Pesticides, which are essentially used in modern agriculture to increase the crop yield, may intensify the environmental pollution by either retaining in the surface of soil or diffusing into surface or ground water [69]. Therefore, the removal of pesticide residues from the aquatic environment is very important to protect the environment [70,71]. Diazinon is an organophosphorus insecticides which is considered in the second category (moderately hazardous) in terms of risks by the World Health Organization (WHO); which has a great effect on the destruction of various types of crawling insects, flying insects, spiders and acarians. Diazinon is one of the nerve poisons that has destructive effects on humans and animals. In general, this compound leads to the inhibition of the acetylcholinesterase enzyme and thus threatens the life of humans and animals [72]. According to The European Union, the maximum allowed amount of pesticides and individual compounds in drinking water should not exceed 0.5 μg/L and 0.1 μg/L, respectively [73,74].

Chitosan (CS) is a natural polysaccharide that is placed in the second place after cellulose in terms of abundance in nature. Chitosan is produced during the process of alkaline N-deacetylation of chitin [75,76]. Many applications of chitosan have been reported in different scientific fields, one of which is wastewater treatment through adsorption process. Therefore, many research studies have been presented in the field of bio-adsorbent chitosan and its composites in the removal of pollutants [77,78].

Boehmite nanoparticles (BNPs) with the formula AlOOH is a group of hydrated aluminum oxide that has high thermodynamic stability [[79], [80], [81]]. BNPs also have high surface area which makes them suitable as an important industrial material for the preparation of catalysts and adsorbents. The point of zero charge (PZC) of boehmite is between 7.7 and 9.4, so it probably exists in nature with positive charges on its surface. Therefore, the protonated surface of boehmite attracts negatively charged compounds through electrostatic attraction [82,83].

Ethylenediaminetetraacetic acid (EDTA) is widely used in different coordination applications as a chelating agent [84,85]. Upon chelating with 5-membered rings, the adsorbent coordinates with a water molecule and a free carboxyl group. This coordinating, and hence chelating, ability of EDTA with different metal ions and adsorbates (coordination number of 6) makes it suitable for different applications such as metal ion remediation, paper industry, synthetic detergents, and pollutant adsorption [86,87].

**Scheme 1:**
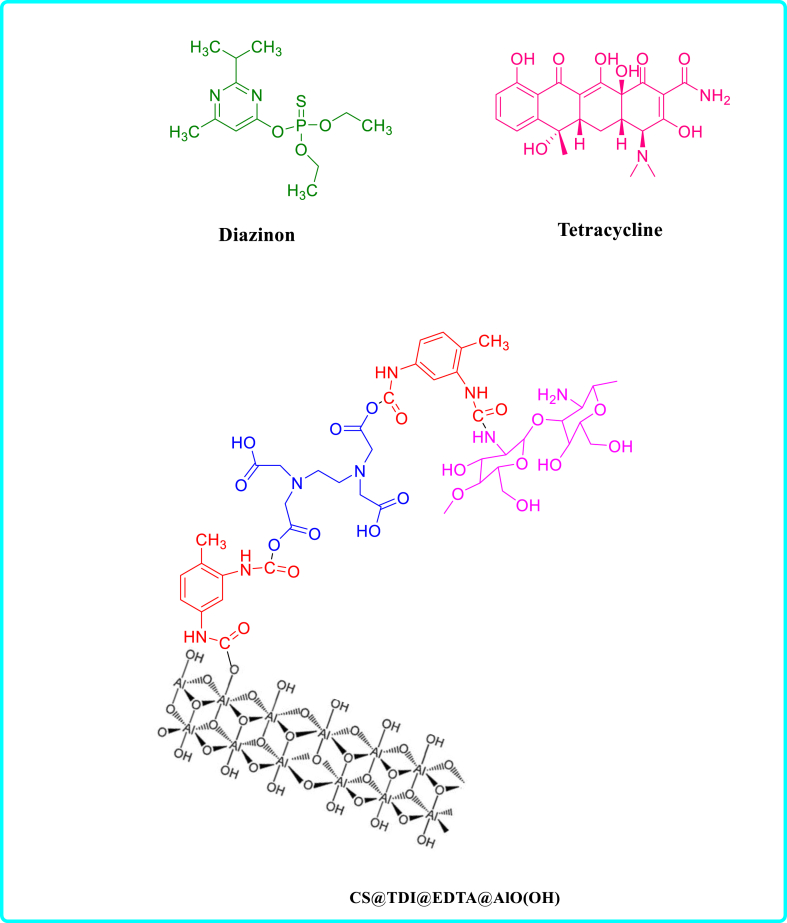
structure of diazinon, tetracycline and CS@TDI@EDTA@γ-AlO(OH).

This work presents a novel and effective adsorbent (CS@TDI@EDTA@γ-AlO(OH)) prepared using three components: chitosan, BNPs and EDTA using amide and ester bridges. This adsorbent contains hydroxyl, amine, and carboxyl groups as chelating agents, which gives it an exceptional adsorption capability. For this purpose, an eco-friendly and simple method was used. Then, the synthesized compound was used as a strong adsorbent to remove diazinon poison and tetracycline antibiotic. CS@TDI@EDTA@γ-AlO(OH) benefits from easy preparation, available and green raw materials, high adsorption capacity, and reusability. Therefore, it outperforms other adsorbents reported previously. The performance of the prepared CS@TDI@EDTA@γ-AlO(OH) was examined by changing the effective factors such as removal temperature and pH, concentration, and adsorbent dose. The adsorption kinetics and isotherms of the prepared CS@TDI@EDTA@γ-AlO(OH) were also explored to investigate the effect of the prepared CS@TDI@EDTA@γ-AlO(OH) on the adsorption of diazinon and tetracycline (Scheme 1).

## 2 Experimental

### 2.1 Chemicals

All the chemicals supplied from Aldrich or Merck Companies were of high purity and used without further processing. Diazinon (98.2 %) was purchased from Merck Co. while tetracycline (99 %) was supplied from Darou Pakhsh Pharmaceutical Manufacturing Co., Tehran, IRAN. Characterization of CS@TDI@EDTA@γ-AlO(OH) was performed by FTIR (Shimadzu 8400 s), FESEM (TESCAN-MIRA3), EDX (Numerix DXP-X10P), and TGA (Bahr Company STA 504) analyses. XRD patterns of the CS@TDI@EDTA@γ-AlO(OH) were recorded using TW 1800 diffractometer (*λ*_CuKa_ = 1.54050 Å).

### 2.2 Preparation of γ-AlO(OH)

A dispersion of 10 g of Al(NO_3_)_3_⋅9H_2_O in distilled water (15 ml) was added dropwise to NaOH aqueous solution (0.003 M) to obtain a mixture with cream color which was then ultrasonicated at 25 °C for 3 h. The desired BNPs (γ-AlO(OH)**)** were obtained after filtering the solution under vacuum, washing the filtrate with water, and then drying the solid material at 220 °C for 4 h [88].

### 2.3 Synthesis of CS@TDI@EDTA@γ-AlO(OH)

**Scheme 2:**
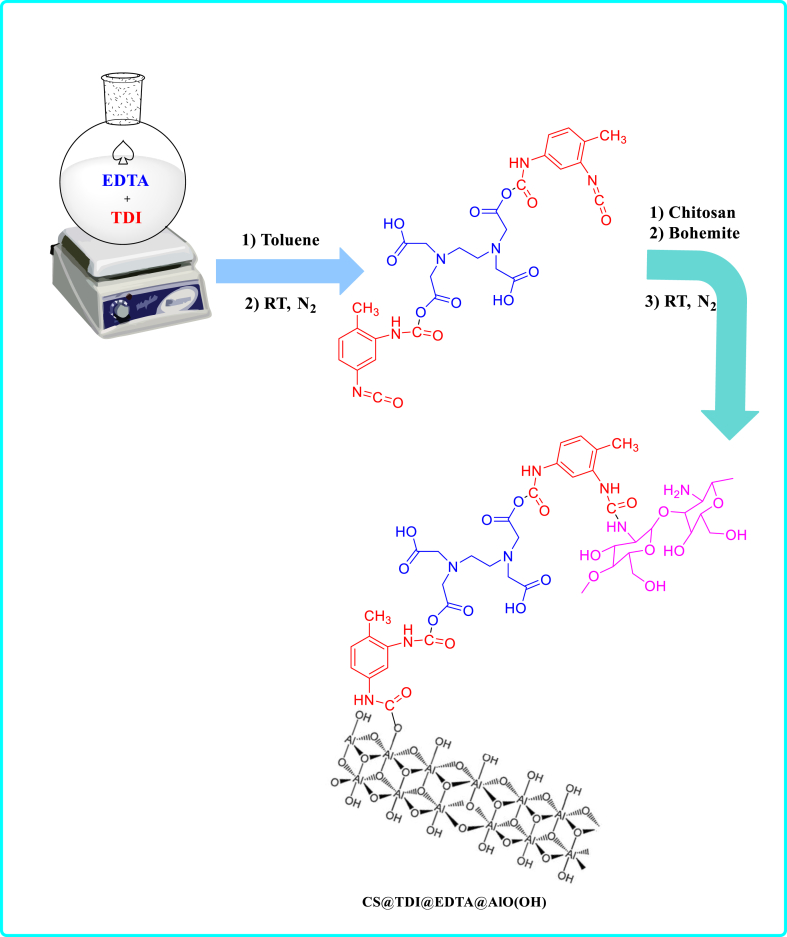
Schematic diagram of CS@TDI@EDTA@γ-AlO(OH) preparation.

First, ethylenediaminetetraacetic acid (EDTA, 0.29 g) was dispersed in the toluene (10 ml) under stirring for 15 min at ambient temperature. After dropwise addition of toluene diisocyanate (TDI, 0.5 ml), stirring was performed under nitrogen at room temperature for 24 h. Then, the mixture was stirred at room temperature for another 24 h after the addition of chitosan (0.1 g) and boehmite (0.1 g). Finally, a white precipitate (CS@TDI@EDTA@γ-AlO(OH)) was obtained after filtering, washing twice with toluene and EtOH, and then drying for 8 h at 80 °C (Scheme 2).

## 3 Characterization of CS@TDI@EDTA@γ-AlO(OH)

Characterization of the synthesized CS@TDI@EDTA@γ-AlO(OH) adsorbent was performed by applying XRD, FTIR, TGA, FESEM, and EDX techniques.

**Fig. 1:**
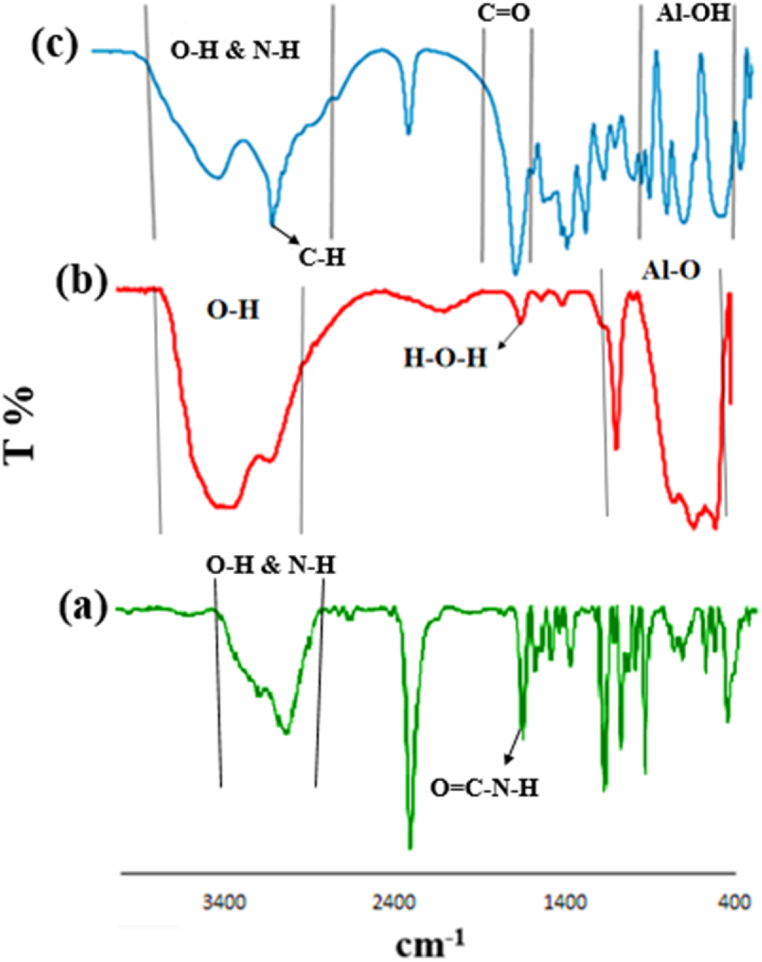
FTIR spectra obtained for (**a**) chitosan, (**b**) BNPs, and (**c**) CS@TDI@EDTA@γ-AlO(OH).

FTIR spectra of the chitosan (**a**), BNPs **(b)** CS@TDI@EDTA@γ-AlO(OH) (**c**) are shown in Fig. 1 according to **schematics 1** and **2**. FT-IR spectra of chitosan, adsorption bands at 3450-2900 cm^−1^ are associated with the stretching vibration of O–H and N–H bonds. In addition, the adsorption peak at 1650 cm^−1^ corresponds to C=O bonds in the amide (Fig. 1a). In the case of BNPs, the adsorption peaks at 3090 cm^−1^ and 3310 cm^−1^ can be assigned to the O–H groups on the surface of BNPs. The adsorption bands at 480 cm^−1^, 605 cm^−1^, and 735 cm^−1^ are related to Al–O bond. In addition, the peaks at 1164 cm^−1^ and 1069 cm^−1^ are attributed to the surface H–*O*–H bonds [88] (Fig. 1b). The adsorption bands at 3578-2500 cm^−1^ are resulted from the O–H stretching vibration in hydroxyl groups of the carboxylic acid. The peaks of N–H and O–H bonds are located under this broad peak. The adsorption peak at 3017 cm^−1^ belongs to the C–H in aromatic compounds. In addition, the adsorption peak at 1700 cm^−1^ is related to C=O bond in the carboxylic acid. Also, peaks related to C=O groups of the amide and ester overlap with the peak width of 1650–1740 cm^−1^ region. Vibrations of Al–OH bond can be observed between 800 and 564 cm^−1^ (Fig. 1c).

**Fig. 2:**
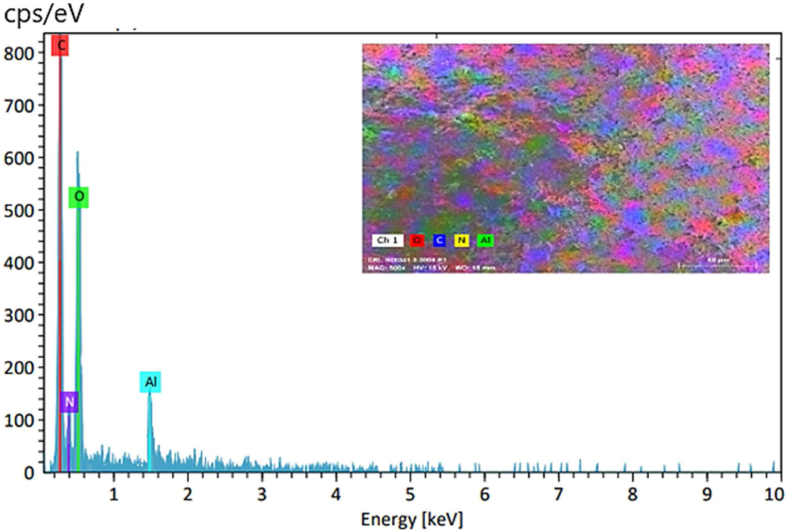
Elemental composition of the synthesized CS@TDI@EDTA@γ-AlO(OH) obtained by EDX technique.

EDX analysis is an analytical technique used to characterize elemental composition and chemical analysis. According to the EDX analysis (Fig. 2), it is clear that the CS@TDI@EDTA@γ-AlO(OH) is composed of C (38.24 %), O (42.00 %), N (14.37 %) and Al (5.39 %) elements, confirming the successful synthesis of the CS@TDI@EDTA@γ-AlO(OH) composite.

**Fig. 3:**
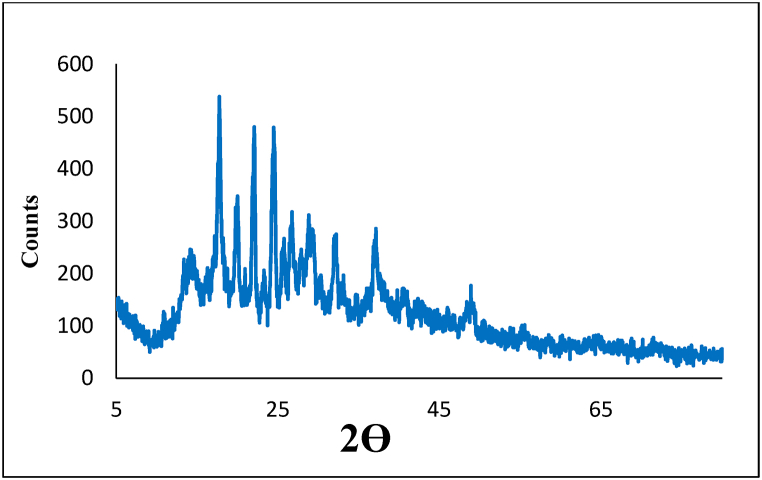
Chemical investigation of the CS@TDI@EDTA@γ-AlO(OH) by XRD technique.

According to the XRD analysis, the adsorbent is composed of chitosan, EDTA and boehmite compounds (JCPDS card Nos. 00–0391894, 00-008-0659 and 00-003-0065, respectively) (Fig. 3). The peaks at 2θ of 13.84°, 17.74°, 20.03°, 22°, 37.15° and 53.92° confirm the presence of BNPs. Also, the peaks at 2θ of 14.28°, 19.98°, 23.40°, 24.60°, 26.43°, 26.75°, 28.54°, 28.82°, 33.13°, 39.78°, 41° and 48.90° are related to EDTA and other components of the adsorbent.

**Fig. 4:**
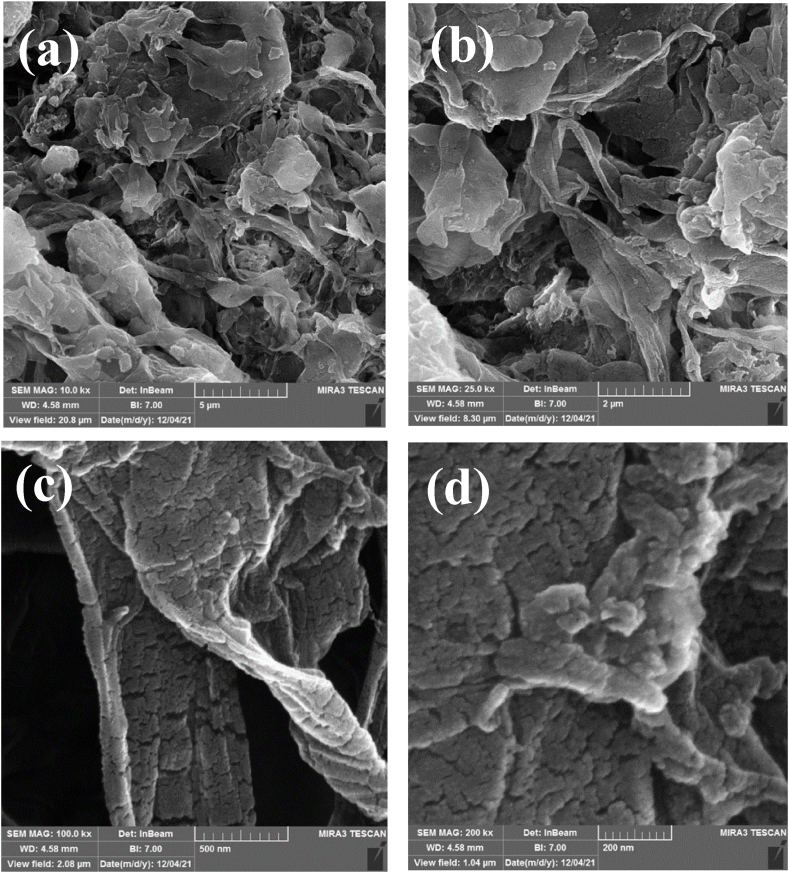
FESEM images of the CS@TDI@EDTA@γ-AlO(OH).

Fig. 4 shows the FESEM images of the synthesized CS@TDI@EDTA@γ-AlO(OH). Since FESEM images are used to investigate and observe the morphology and surface of materials. The FESEM images of the adsorbent clearly show that chitosan sheets have changed form from regular and uniform to irregular and intertwined sheets that have a non-uniform distribution (Fig. 4a–d). These existing entanglements are due to the composite of boehmite and chitosan nanoparticles with other components used to prepare the desired adsorbent. Also, the images **c** and **d** clearly show the mesoporousness of the surface of the structure. Therefore, by observing the change in the morphology of the raw components used in the adsorbent structure compared to the final structure, along with other analyzes provided to confirm the prepared structure, it confirms the success of the adsorbent preparation [[89], [90], [91]].

**Fig. 5:**
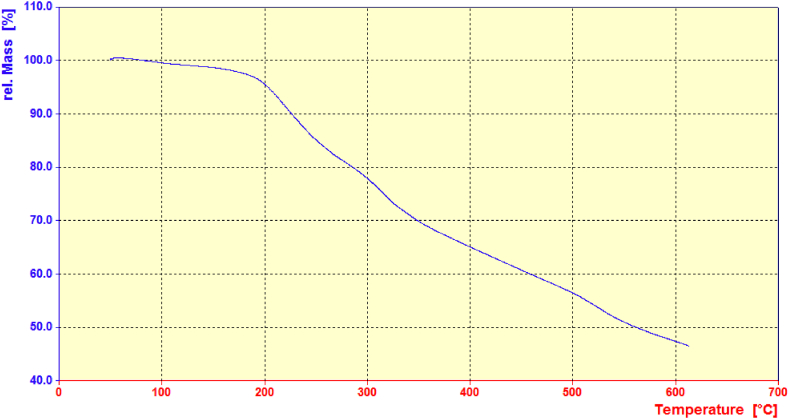
TGA curve of the CS@TDI@EDTA@γ-AlO(OH).

According to the thermogravimetric analysis (TGA) of the CS@TDI@EDTA@γ-AlO(OH) (Fig. 5), there are two weight loss peaks at 190–330 °C and 350–600 °C. The first peak is resulted from the destruction of the organic compounds in the adsorbent (i.e., chitosan and TDI) [92] while the second peak is related to the inorganic boehmite in the structure. The gentle slope of the weight loss diagram may be resulted from the organic compounds grafted onto the surface of the boehmite [88].

## 4 Adsorption tests

The removal of diazinon and tetracycline on the surface of CS@TDI@EDTA@γ-AlO(OH) composite was examined by varying the experimental conditions including adsorbent dosage as well as the adsorption temperature, time and pH. After each experiment, the adsorbent was filtered from the solution and the concentration of diazinon and tetracycline was measured using UV–vis spectrophotometer. Eq. (1) can be used to calculate the removal percentage of diazinon and tetracycline while Eq. (2) yields the adsorption capacity (q_t_ in mg/g) of the adsorbent, respectively:

(1) $$\text{Removal}\;\%=\frac{\left(C_{0}-\;C_{e}\right)\;}{C_{0}}\times 100\;$$

(2) $$\text{qe}=\frac{\left(C_{0}-\;C_{e}\right)\;V}{W}$$

where C_o_ (mg/L) is the initial concentration of diazinon and tetracycline, Ce (mg/L) is the equilibrium concentration of diazinon and tetracycline, V (L) is the volume of diazinon and tetracycline solution, and W (g/L) is the adsorbent concentration.

### 4.1 The influence of initial concentration of diazinon and tetracycline

**Fig. 6:**
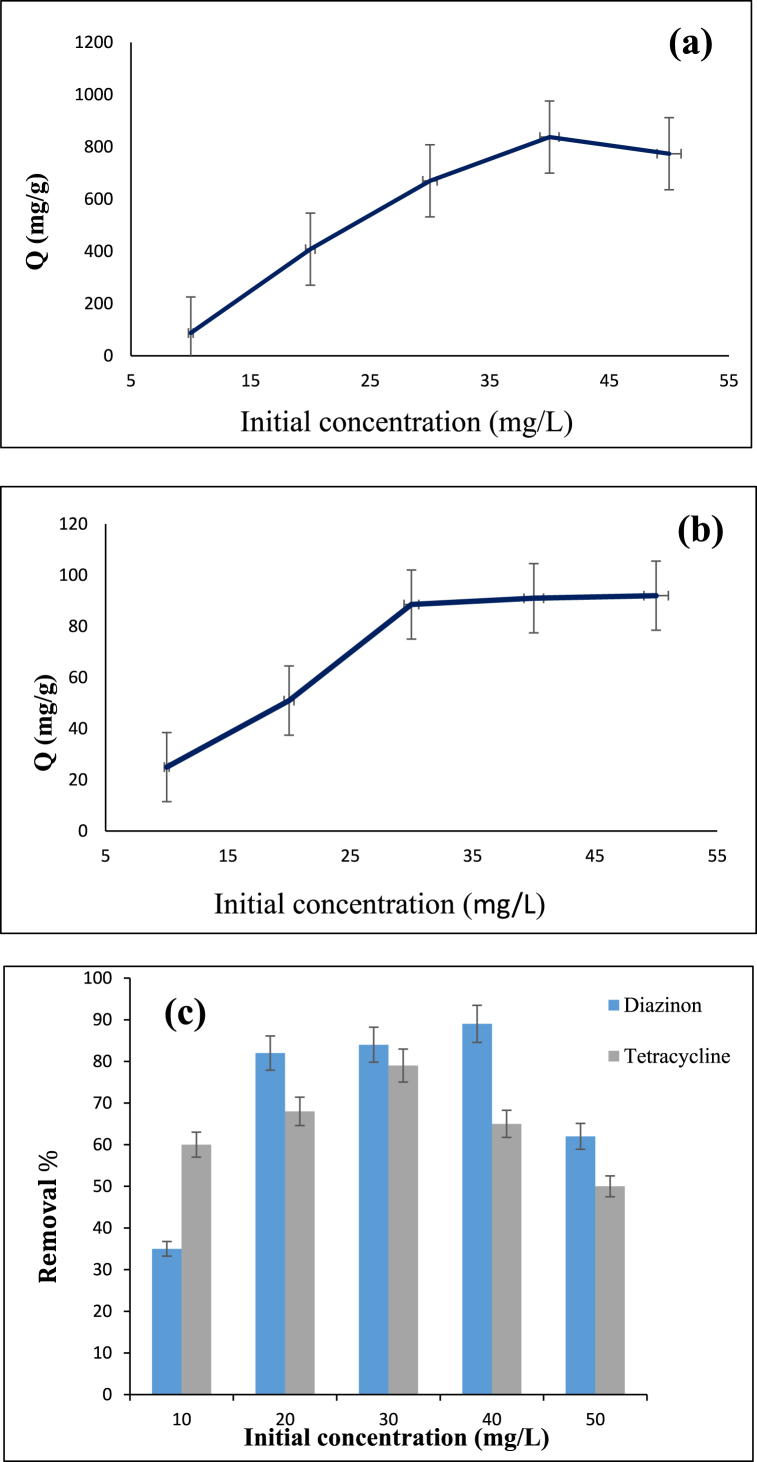
Dependency of adsorption capacity on the initial concentration of (**a**) diazinon, (**b**) tetracycline. (**c**) removal percentage of diazinon and tetracycline.

The effect of initial concentration of diazinon and tetracycline was investigated by mixing 0.12 g/L of adsorbent with a solution (100 ml) containing 10–50 mg/L diazinon and tetracycline at pH 7 and 60 °C for 120 min. After filtering the solutions, UV–vis spectrophotometer was applied to measure the adsorbance. According to the results obtained for diazinon and tetracycline (Fig. 6a and **b**, respectively) the optimal concentration for diazinon was 40 mg/L while that for tetracycline was 30 mg/L. Also, in Fig. 6c, the graph of the percentage removal of diazinon and tetracycline contaminants using the prepared adsorbent is presented. As can be seen, for the optimal concentration of diazinon, the percentage of removal is 89 % and the percentage of tetracycline removal at the optimal concentration is 79 %.

### 4.2 The influence of adsorbent concentration

**Fig. 7:**
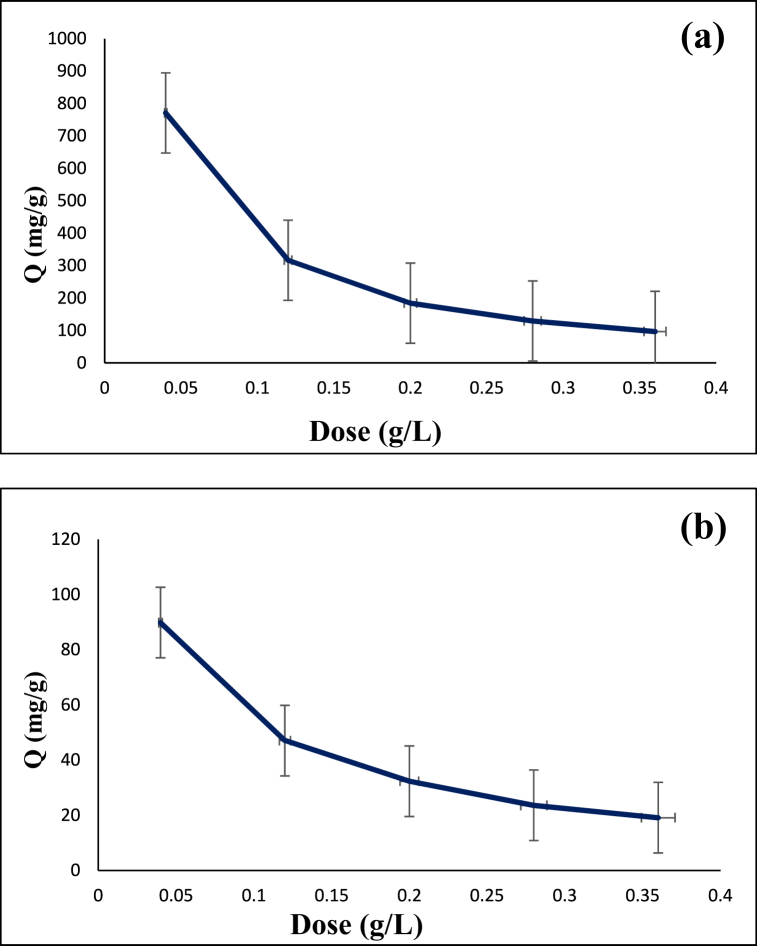
Dependency of adsorption capacity on the adsorbent concentration: (**a**) diazinon, and (**b**) tetracycline.

The dependency of adsorption capacity of the CS@TDI@EDTA@γ-AlO(OH) composite on the adsorbent concentration (0.04–36 g/L) for the removal of diazinon and tetracycline was studied. Fig. 7 shows a decrease in the removal of diazinon and tetracycline by increasing the adsorbent concentration from 0.04 to 36 g/L. This can be caused by (i) agglomeration of the adsorbent particles, (ii) insufficient vacancies, and (iii) unchanged specific surface area.

### 4.3 The influence of solution pH

**Fig. 8:**
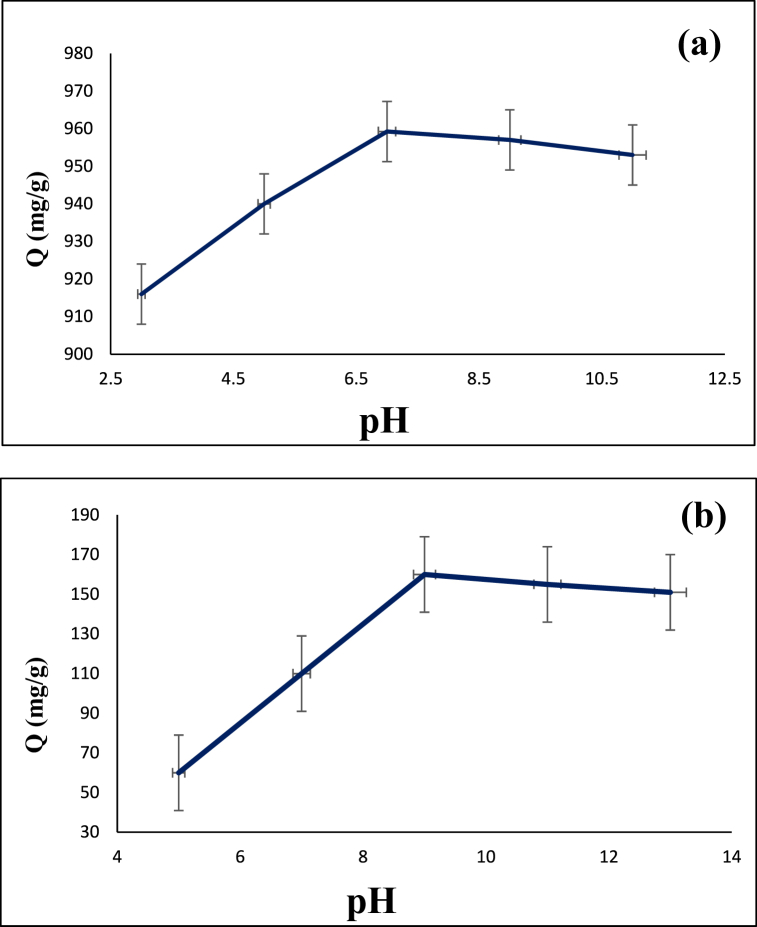
Dependency of adsorption capacity on the pH: (**a**) diazinon, and (**b**) tetracycline.

The solution pH can affect the adsorption capacity. Fig. 8 indicates the dependency of diazinon and tetracycline adsorption on the solution pH. According to the figure, the highest diazinon adsorption capacity was obtained at pH 7 while the maximum tetracycline adsorption capacity was achieved at pH 9. The latter can be caused by the higher negative charge on the surface of the composite mainly due to the deprotonation of surface functional groups. Therefore, stronger electrostatic attraction is created on the surface, leading to higher adsorption capacity.

### 4.4 The influence of temperature

**Fig. 9:**
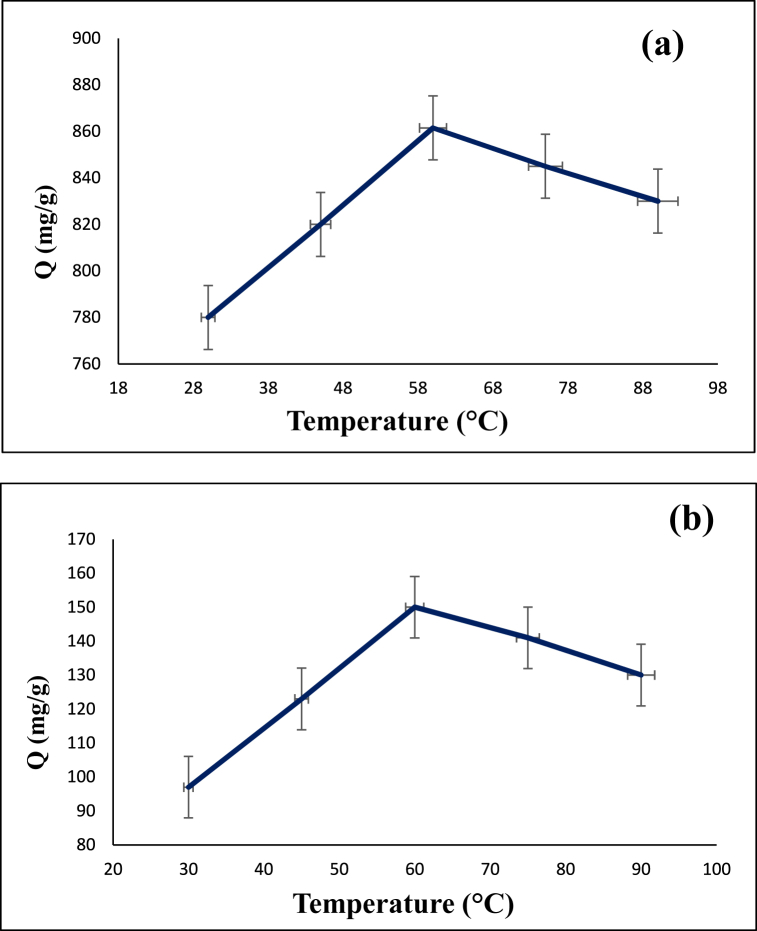
Dependency of adsorption capacity on the temperature: (**a**) diazinon, and (**b**) tetracycline.

The dependency of adsorption capacity of CS@TDI@EDTA@γ-AlO(OH) on the temperature (30, 45, 60, 75 and 90 °C) for removal of diazinon and tetracycline is shown in Fig. 9. It can be seen that for adsorption of both diazinon and tetracycline, higher adsorption capacity is obtained as the temperature increases from 30 °C to 60 °C. However, the adsorption capacity weakens by further rising the temperature from 60 °C to 90 °C. This indicates that the adsorption is an exothermic process. Therefore, the optimal temperature for the adsorption of both diazinon and tetracycline is 60 °C.

### 4.5 The influence of time

**Fig. 10:**
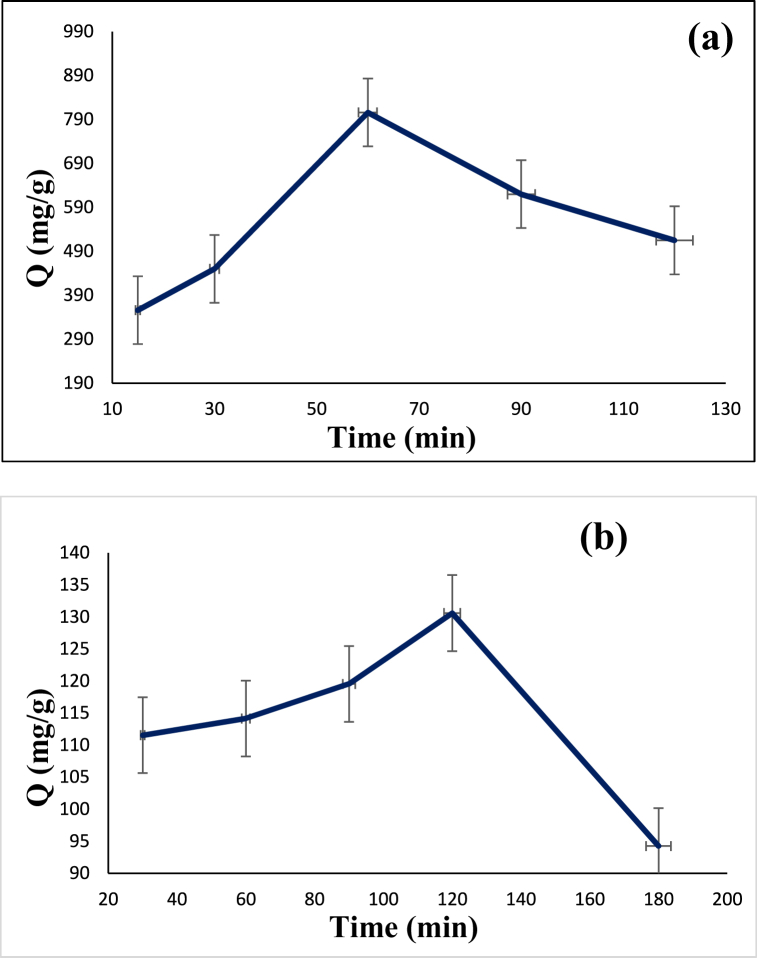
Dependency of adsorption capacity on time: (**a**) diazinon, and (**b**) tetracycline.

The adsorption capacity was investigated for both diazinon and tetracycline in the presence of 0.12 g/L of the synthesized adsorbent. For the diazinon adsorption, pH was adjusted at 7 while for tetracycline the pH was 9. The tests were performed at different times of 15, 30, 60, 90, and 120 min in the case of diazinon and 30, 60, 90, 120, and 180 min in the case of tetracycline. The equilibrium time to achieve the highest adsorption of diazinon and tetracycline was 60 min and 120 min, respectively (Fig. 10). Beyond these optimum values, adsorption capacity decreases which may be related to the lack of sufficient adsorption sites on the surface of the adsorbent. Therefore, the time values of 60 min and 120 min were selected as the optimal contact time for the adsorption of diazinon and tetracycline, respectively.

### 4.6 Kinetics study

**Fig. 11:**
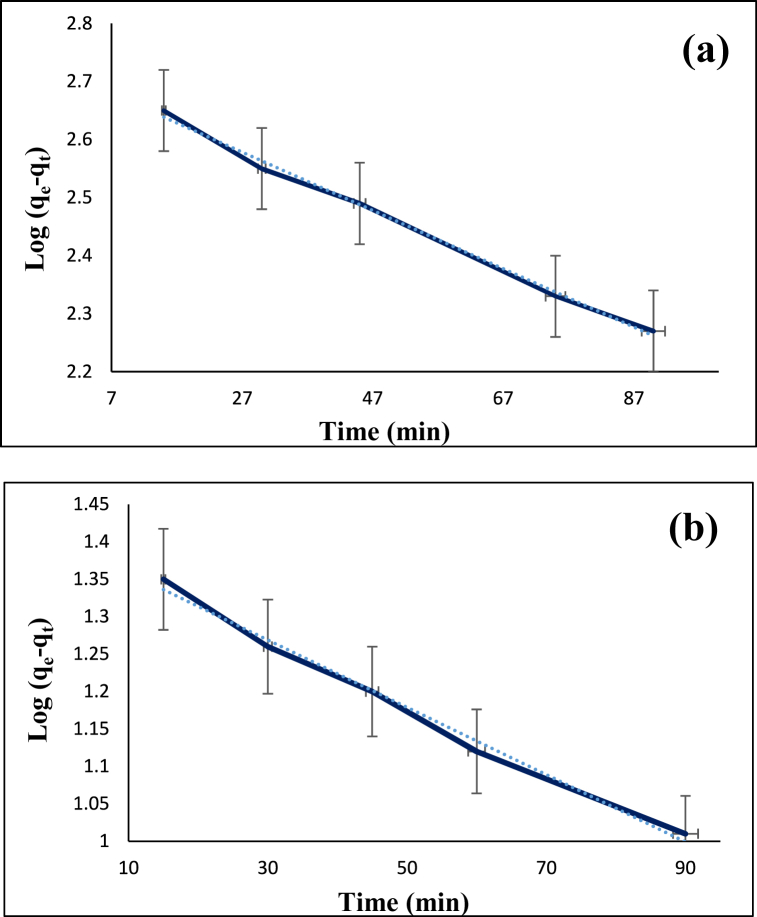
Pseudo-first-order model for the adsorption of diazinon (**a**) and tetracycline (**b**) on CS@TDI@EDTA@γ-AlO(OH).

**Fig. 12:**
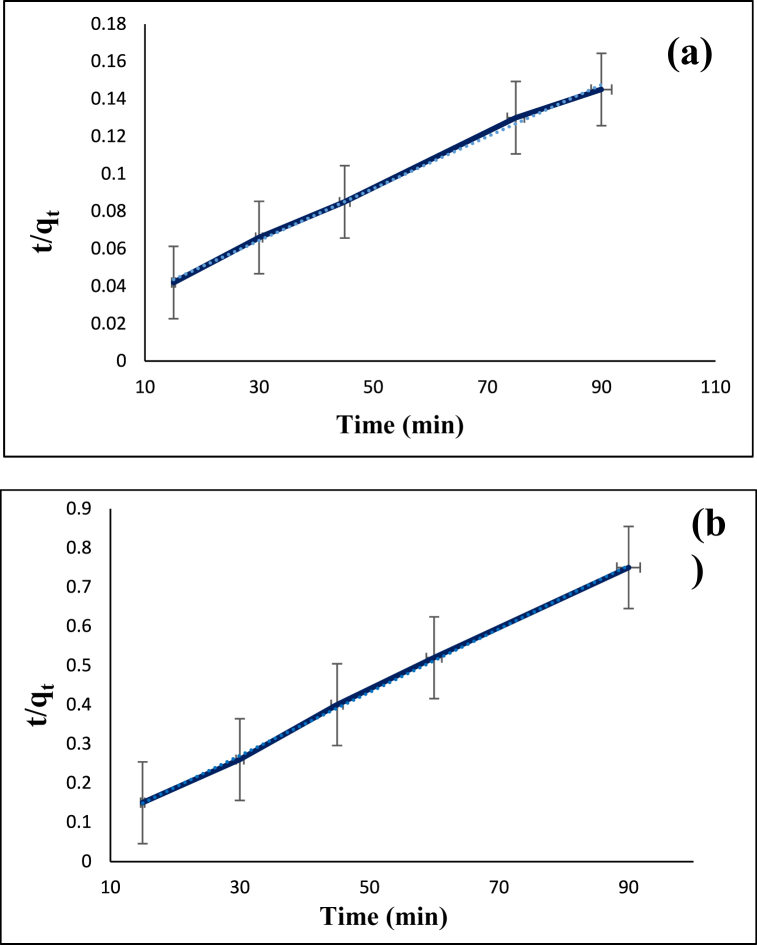
Pseudo-second-order model for the adsorption of diazinon (**a**) and tetracycline (**b**) on CS@TDI@EDTA@γ-AlO(OH).

The adsorption efficiency can be investigated by studying the adsorption kinetics which defines the rate of solute adsorption and the time that the adsorbates reside at the interface between solid and liquid. The adsorption velocity is defined as the number of particles that are adsorbed on the surface of the adsorbent per second. In this study, the adsorption kinetics of the diazinon and tetracycline was examined by using 0.12 g/L adsorbent at 60 °C. In the case of diazinon, the pH and the initial concentration were 7 and 40 mg/L while these parameters for tetracycline were 9 and 30 mg/L, respectively. The stirring time for diazinon was 15, 30, 45, 75, 60, and 90 min while that for tetracycline was 15, 30, 45, 60, and 90 min. After that, the samples were filtered. The kinetic data obtained for the adsorption of diazinon and tetracycline was examined by applying pseudo-first-order (Eq. (3)) and pseudo-second-order (Eq. (4)) models,

(3) $$\log\left(q_{e}-q_{t}\right)=\log\;q_{e}-\left(\frac{K_{1}}{2.303}\right)t$$

(4) $$\frac{t}{q_{t}}=\frac{1}{K_{2\;}q_{e}^{2}}+\left(\frac{1}{q_{e}}\right)t\;$$

where K_1_ (1/min) is the rate constant for the first-order adsorption kinetic model, K_2_ (g/mg.min) is the rate constant for the second-order adsorption kinetic model, q_t_ (mg/g) is the adsorption capacity in time t, and qe (mg/g) is the adsorption capacity at equilibrium. Fig. 11, Fig. 12 as well as Table 1 show the related parameters for these kinetic models. For the adsorption of diazinon and tetracycline, pseudo-second-order model yielded R^2^ values of 0.9970 and 0.9988, respectively. Also, the coefficient k for diazinon and tetracycline was 0.000085 and 0.0023, respectively. In any kinetic model, if the correlation factor (R^2^) is larger and the rate constant (K_t_) is smaller, it is more favorable and the adsorption mechanism follows it. These results indicate that the adsorption of diazinon and tetracycline follows pseudo-second-order kinetics model. Also, error analysis in kinetic for diazinon (**a**) and tetracycline (**b**) on CS@TDI@EDTA@γ-AlO(OH) are shown in Table 2.

**Table 1:** Kinetic parameters for the adsorption of diazinon (**a**) and tetracycline (**b**) on CS@TDI@EDTA@γ-AlO(OH) in different models.

Model	Parameter	Diazinon	Tetracycline
Pseudo-first-order	**q**_**e**_**(mg/g)**	517.84	25.33
	**k**	0.01	0.0103
	**R**^**2**^	0.9956	0.9914
Pseudo-second-order	**q**_**e**_**(mg/g)**	714.28	123.45
	**k**	0.000085	0.0023
	**R**^**2**^	0.9970	0.9988
Intraparticle diffusion	**k**_**i**_	80.24	2.105
	**C**	726	128.49
	**R**^**2**^	0.9842	0.9764
Bangham model	**k**_**0**_	0.014	0.0047
	**α**	0.4804	0.2445
	**R**^**2**^	0.9192	0.9896
Boyd model	**R**^**2**^	0.9761	0.9365

**Table 2:** Error analysis in kinetic for the adsorption of diazinon and tetracycline on CS@TDI@EDTA@γ-AlO(OH).

Model	Parameter	Diazinon	Tetracycline
Pseudo-first-order	**RMSE**	0.0509	0.0255
	**ERRSQ**	0.2372	0.1702
Pseudo-second-order	**RMSE**	0.0238	0.0144
	**ERRSQ**	0.1140	0.0656
Intraparticle diffusion	**RMSE**	0.0737	0.0496
	**ERRSQ**	0.6199	0.4671
Bangham model	**RMSE**	0.2341	0.3416
	**ERRSQ**	0.7923	0.5419

#### 4.6.1 Intraparticle diffusion model

The intraparticle diffusion model was applied to analyze the kinetic data in order to investigate the mechanism of diazinon and tetracycline adsorption on the surface of CS@TDI@EDTA@γ-AlO(OH). This model can be defined by Eq. (5). The related parameters for the adsorption of diazinon and tetracycline on the CS@TDI@EDTA@γ-AlO(OH) are given in Table 1.

(5) $$q_{t}=k_{i}t^{1/2}+C\;$$

where k_i_ (mg. min^−0.5^/g) is the rate coefficient and C (mg/g) is the thickness of the boundary layer, respectively [93].

**Fig. 13:**
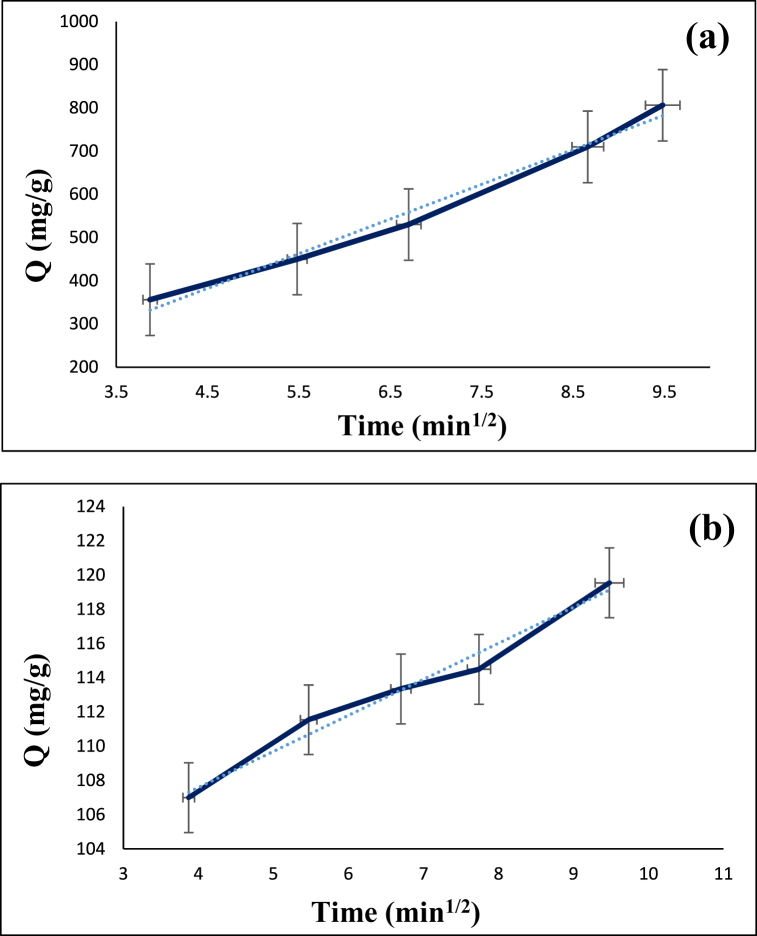
Intraparticle diffusion model for the adsorption of diazinon (**a**) and tetracycline (**b**) on CS@TDI@EDTA@γ-AlO(OH).

As depicted in Fig. 13, the intraparticle diffusion model does not intersect the origin, indicating that intraparticle diffusion is not the only parameter that controls the rate of adsorption of diazinon and tetracycline on the surface of the synthesized adsorbent. The calculated intraparticle diffusion rate constant (K_ip_) for the adsorption of diazinon and tetracycline was 80.24 and 2.105 mg min^−0.5^/g, respectively [94].

**Fig. 14:**
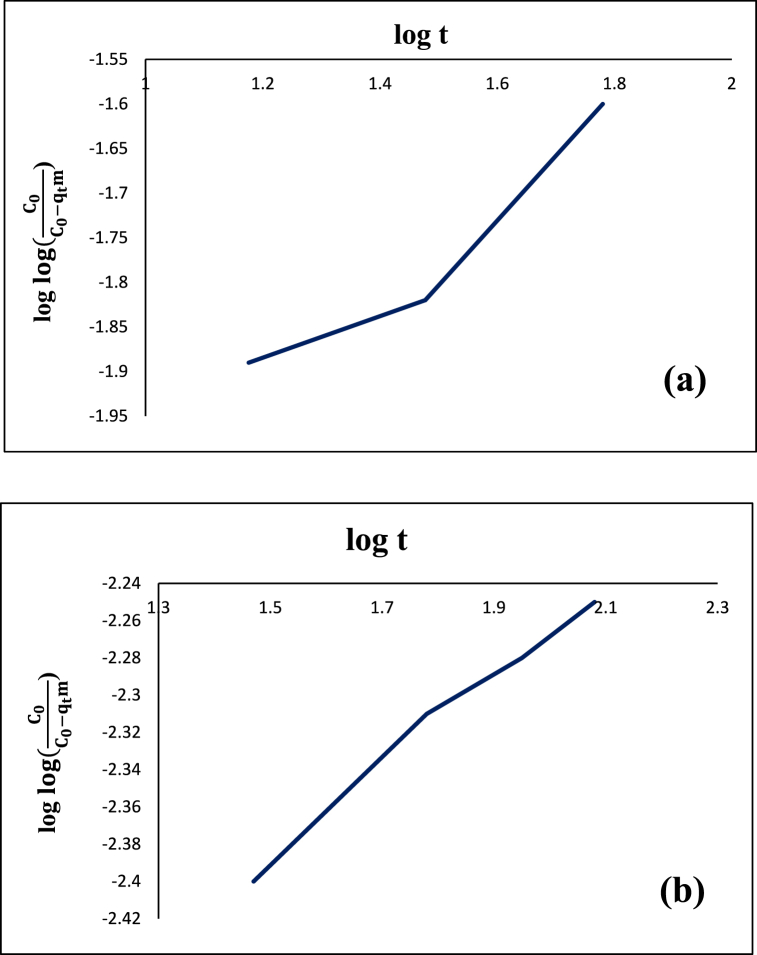
Bangham model of diazinon (**a**) and tetracycline (**b**) on CS@TDI@EDTA@γ-AlO(OH).

In the case where only pore diffusion controls the adsorption rate, Bangham model (Fig. 14) as defined by Eq. (6) can be applied,

(6) $$l\text{og}\left\{\log(\frac{C_{0}}{C_{0}{-q}_{t}m}\right\}=\log\left(\frac{k_{0}m}{2.303V}\right)+\alpha \log\;t$$

where C_0_ (mg/L) is the initial solute concentration, V (ml) is the solution volume, m is the adsorbent mass in 1 L of the solution, qt (mg/g) is the adsorption capacity at time t, and k_o_ and α are constant values.

In order to predict the parameter that actually controls the rate of adsorption process, Boyd plot is used as expressed by Eq. (7),

(7) $$B_{t}=-0.4977-\mathrm{Ln}\;\left(1-F\right)$$

where F is the fraction of adsorbed solute, and B_t_ is the mathematical function of F at time t.

### 4.7 Adsorption isotherms

The optimum utilization of an adsorbent depends on its interaction the adsorbate, the stability of which can be determined by adsorption isotherms. Their shape is also indicative of the affinity of the molecules for adsorption. In this study, Langmuir (Eq. (8)), Freundlich (Eq. (9)), and Temkin (Eq. (10)) isotherms, which are different mathematical forms of adsorption isotherms, were applied to further investigate the adsorption of diazinon and tetracycline on CS@TDI@EDTA@γ-AlO(OH).

(8) $$\frac{C_{e}}{q_{e}}=\frac{1}{K_{L}q_{m}}+\frac{1}{q_{m}}C_{e}$$

(9) $$\ln\;q_{e}=\ln\;K_{F}+\left(\frac{1}{n}\right)\ln\;C_{e}$$

(10) $$q_{e}=B_{T}\;\mathrm{Ln}\left(K_{T}\right)+B_{T}\;\ln\;C_{e}$$

**Fig. 15:**
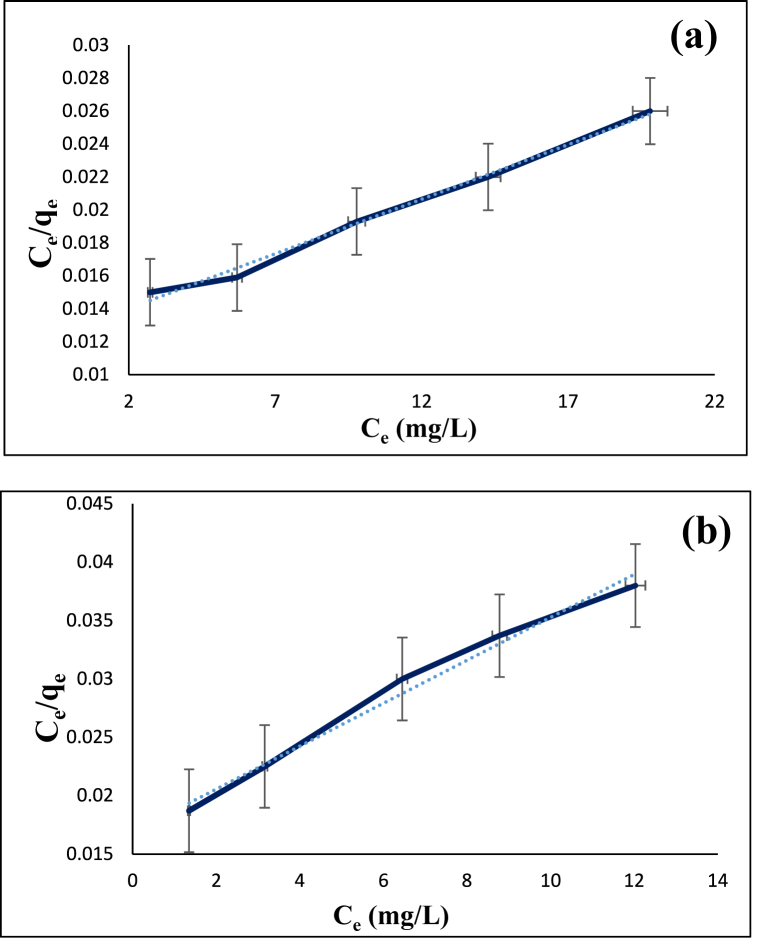
Langmuir isotherm model for the adsorption of diazinon (**a**) and tetracycline (**b**) on CS@TDI@EDTA@γ-AlO(OH).

**Fig. 16:**
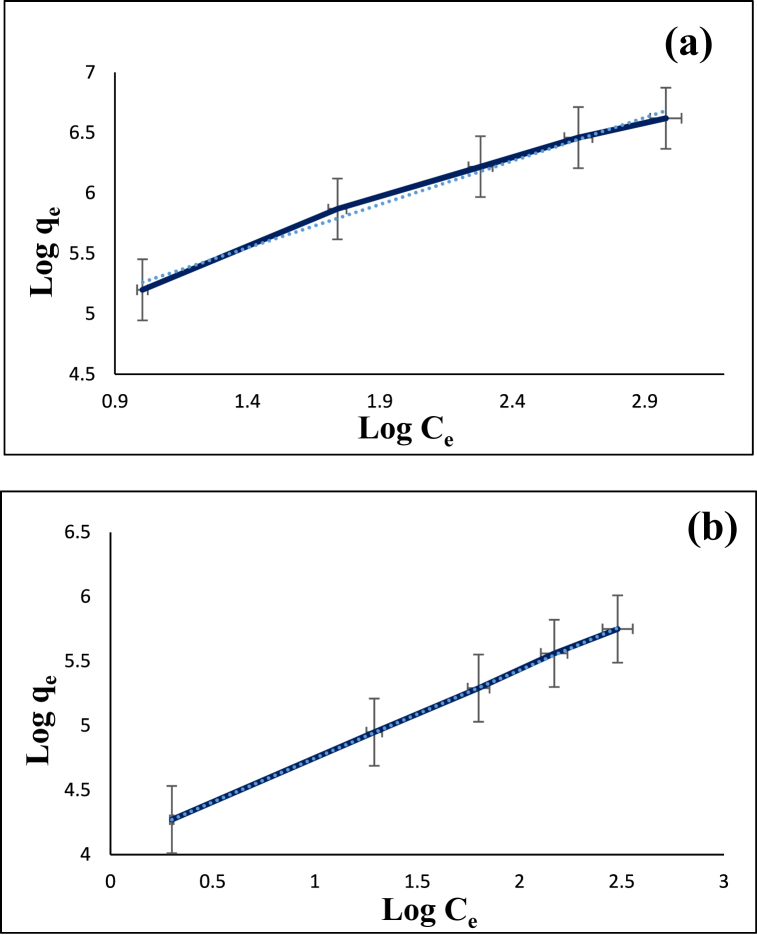
Freundlich isotherm model for the adsorption of diazinon (**a**) and tetracycline (**b**) on CS@TDI@EDTA@γ-AlO(OH).

**Fig. 17:**
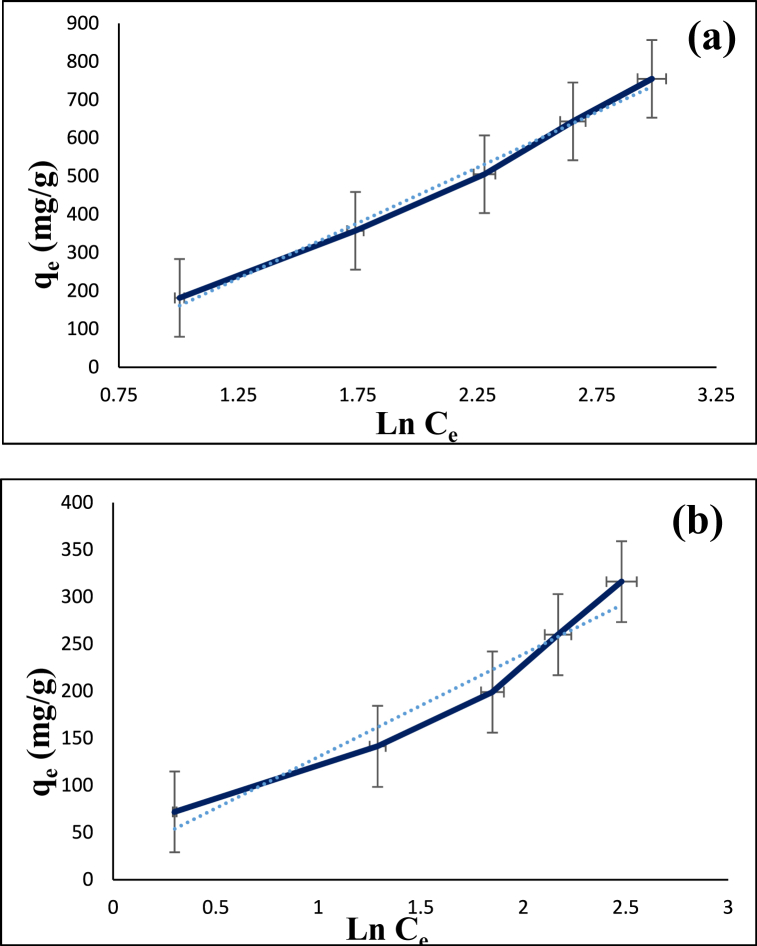
Temkin isotherm model for the adsorption of diazinon (**a**) and tetracycline (**b**) on CS@TDI@EDTA@γ-AlO(OH).

In these equations, C_e_ (mg/L) is the concentration of diazinon or tetracycline, K_F_ (mg/g) is the Freundlich constant related to the adsorption capacity, n is the Freundlich constant corresponding to the adsorption intensity, B is Temkin constant, and K_T_ (L/mg) is the equilibrium binding constant. The comparison of the experimental data with Langmuir, Freundlich and Temkin isotherms is presented in Fig. 15, Fig. 16, Fig. 17, respectively. In the adsorption isotherms, the higher the R^2^ value, the better fit exists between the experimental data and the adsorption isotherm model. In the case of diazinon, the R^2^ value of the Langmuir model is higher than that of the other two models. Therefore, the Langmuir adsorption model is more suitable for describing the adsorption of diazinon. As shown in Table 3, the maximum adsorption capacity (q_max_) at 60 °C was 1428.5 mg/g. In the case of tetracycline, the adsorption is more consistent with the Freundlich adsorption model with the q_max_ of 555.5 mg/g at 60 °C (Table 3). In addition, Table 4 shows the error analysis in the isotherm for the adsorption of diazinon and tetracycline on CS@TDI@EDTA@γ-AlO(OH).

**Table 3:** Parameters of different isotherm models for the adsorption of diazinon and tetracycline on CS@TDI@EDTA@γ-AlO(OH).

Model	Parameter	Diazinon	Tetracycline
Langmuir	**q**_**max**_**(mg/g)**	1428.5	555.5
	**K**_**L**_	0.068	0.106
	**R**^**2**^	0.99	0.98
Freundlich	**K**_**f**_	93.92	58.39
	**n**	1.39	1.40
	**R**^**2**^	0.98	0.99
Temkin	**R**^**2**^	0.98	0.94
	**B**	289.91	108.97
	**K**_**T**_	0.64	1.21

**Table 4:** Error analysis in isotherm for the adsorption of diazinon and tetracycline on CS@TDI@EDTA@γ-AlO(OH).

Model	Parameter	Diazinon	Tetracycline
Langmuir	**RMSE**	0.0979	0.0728
	**ERRSQ**	0.8249	0.5647
Freundlich	**RMSE**	1.9361	0.3685
	**ERRSQ**	9.0651	3.7078
Temkin	**RMSE**	0.7955	1.6295
	**ERRSQ**	8.1637	10.5356

### 4.8 Error analysis

Performing error analysis is crucial in data analysis to accurately verify the adsorption model. In this study, residual root mean square error (RMSE) as well as sum of the squares of errors (ERRSQ) were used as error functions. The smaller value of the error function is indicative of more accurate curve fitting. RMSE and ERRSQ error functions can be defined as Eq. (11) and Eq. (12), respectively:

(11) $$\text{RMSE}=\sqrt{\frac{1}{n-2}\sum_{i=1}^{n}{\left(Q_{meas}-Q_{calc}\right)}^{2}}$$

(12) $$\text{ERRSQ}=\sum_{i=1}^{n}{\left(Q_{\text{meas}}-Q_{\text{calc}}\right)}^{2}$$

where Q_meas_ and Q_calc_ are the measured and the calculated concentrations, respectively, and n represents the number of observations in the experimental data. Q_calc_ was calculated by the kinetics model.

### 4.9 Thermodynamic study

Eq. (13) (Van't Hoff relationship) and Eq. (14) were used to calculate different thermodynamic parameters including ΔS° (entropy energy), ΔH° (enthalpy energy), and ΔG° (free energy) for the adsorption of diazinon and tetracycline on CS@TDI@EDTA@γ-AlO(OH).

(13) $$\ln\;K_{d}=\frac{\Delta S}{R}-\frac{\Delta H}{\text{RT}}$$

(14) ΔG°=ΔH°−TΔS°

In these equations, T is temperature (K) and R is gas constant. Table 5 lists he calculated thermodynamic parameters for the adsorption of diazinon and tetracycline on CS@TDI@EDTA@γ-AlO(OH). The results confirm exothermic nature of the adsorption of diazinon (ΔH° = -18.72 kJ/mol) and tetracycline (ΔH° = -7.12 kJ/mol). The ΔH° value demonstrates that diazinon and tetracycline are adsorbed physically on CS@TDI@EDTA@γ-AlO(OH) while the ΔS° value indicates that CS@TDI@EDTA@γ-AlO(OH) has high affinity for the adsorption of diazinon and tetracycline.

**Table 5:** The calculated thermodynamic parameters for diazinon and tetracycline adsorption.

Adsorbent	ΔH° (kJ/mol)	ΔS° (J/mol.K)	ΔG° (kJ/mol) 303 318 333 348			
Diazinon	−18.72	36.15	−6.14	−5.79	−5.26	−4.81
Tetracycline	−7.12	−5.55	−4.25	−3.86	−339	−4.95

### 4.10 The influence of anions in natural water

**Fig. 18:**
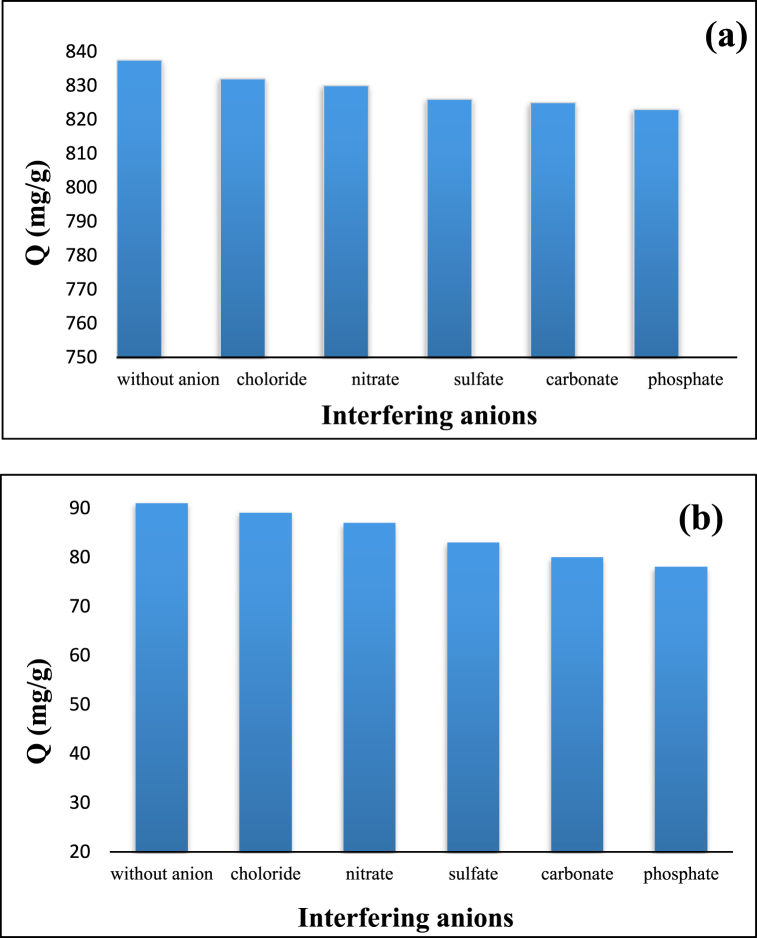
A: Adsorption capacity of diazinon (**a**) and tetracycline (**b**) in the presence of interfering anions. B: Adsorption and desorption of diazinon and tetracycline on the CS@TDI@EDTA@γ-AlO(OH).

Some anions (nitrate, chloride, carbonate, phosphate, and sulfate) that commonly present in natural water may affect the adsorption capacity of diazinon and tetracycline on CS@TDI@EDTA@γ-AlO(OH). To study this interfering effect, high concentrations of the anions (i.e., five times the diazinon and tetracycline) were adopted. According to Fig. 18, the effect of chloride and nitrate anions is negligible, while carbonate, phosphate, and sulfate anions resulted in a slight loss in the adsorption capacity.

### 4.11 The mechanism of adsorption

**Scheme 3:**
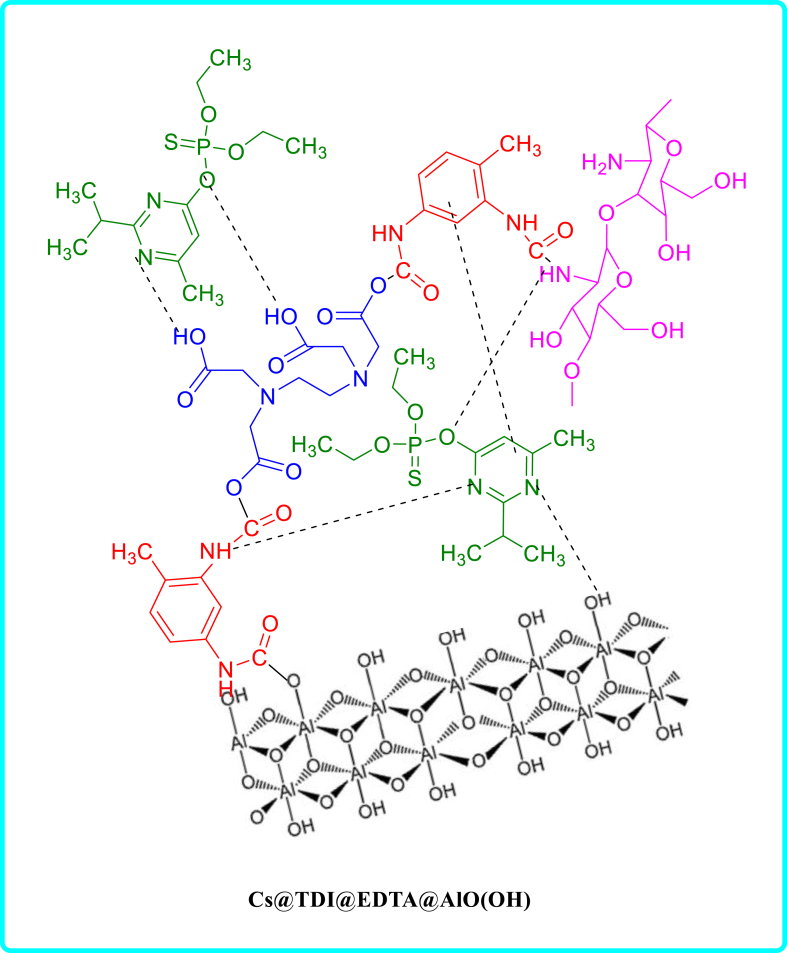
Mechanism of adsorption of diazinon on CS@TDI@EDTA@γ-AlO(OH).

**Scheme 4:**
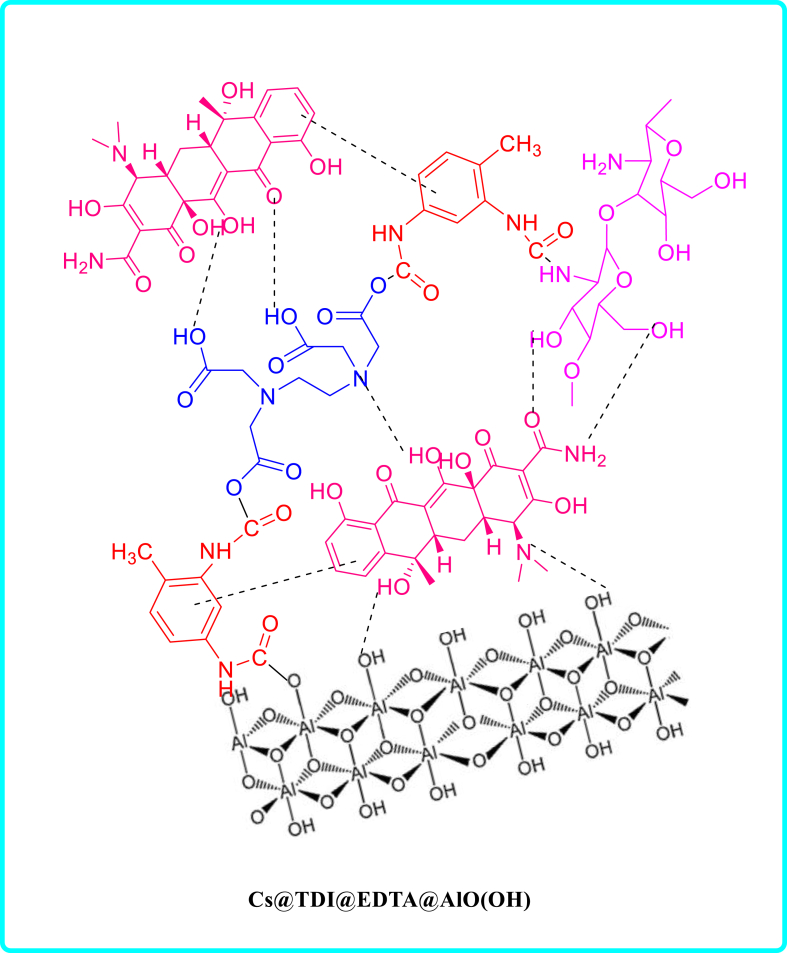
Mechanism of adsorption of tetracycline on CS@TDI@EDTA@γ-AlO(OH).

Scheme 3, Scheme 4 show the mechanism of adsorption for diazinon and tetracycline on CS@TDI@EDTA@γ-AlO(OH). As mentioned earlier, the adsorption of diazinon follows Langmuir isotherm model. Therefore, it takes place by forming a diazinon layer on the surface of the adsorbent. On the other hand, in the adsorption of tetracycline, which is consistent with the Freundlich isotherm model, tetracycline migrates from the solution to the surface of the adsorbent. Also, the boehmite in the structure of the adsorbent has a porous surface that can help the adsorption process by trapping the adsorbent materials in these holes. Three functional groups can be identified in the structure of CS@TDI@EDTA@γ-AlO(OH): carboxylic acid, hydroxyl, and amine. Therefore, hydrogen bonding between electron-rich oxygen, amine groups, and electrostatic interactions may contribute to the adsorption process. In addition, the benzene rings in the composite can adsorb species such as diazinon and tetracycline that contain benzene rings. This adsorption takes place via the π-π interaction.

## 5 Recycling tests

Stability and reusability are two important characteristics of the adsorbents. In order to investigate the reusability of the synthesized CS@TDI@EDTA@γ-AlO(OH) adsorbent for the adsorption of diazinon and tetracycline, it was washed after each adsorption experiment with a mixture of NaCl (0.1 M) and HCl (0.1 M) as cleaning agent. Then, the adsorbent was dried at 80 °C for 6 h to be used in the subsequent adsorption experiment. The adsorption-desorption experiments were repeated four times, the results of which are shown in Fig. 18.

Table 6, Table 7 compare the adsorption properties of CS@TDI@EDTA@γ-AlO(OH) for the adsorption of diazinon and tetracycline, respectively, with those of previously reported adsorbents. The main advantages of the synthesized CS@TDI@EDTA@γ-AlO(OH) in comparison with other adsorbents include easy preparation method, high adsorption capacity, easy removal, reusability, and easy separation.

**Table 6:** Comparison of adsorption ability of CS@TDI@EDTA@γ-AlO(OH) with the adsorbents reported previously for the adsorption of diazinon.

Entry	Adsorbent	Adsorption capacity (mg/g)	adsorbent dosage (mg)	pollutant concentration (mg/L)	Reference
1	Chitosan/Carbon Nanotube	222.86	600	5	[95]
2	chitosan/zero-valent iron nanoparticle	64.93	50	100	[96]
3	NH4Cl-induced activated carbon	250	15	20	[2]
4	Fe_3_O_4_/SiO_2_	206.18	0.2	0.4	[97]
5	**CS@TDI@EDTA@γ-AlO(OH)**	**1428.5**	**3**	**40**	**This work**

**Table 7:** Comparison of adsorption ability of CS@TDI@EDTA@γ-AlO(OH) with the adsorbents reported previously for the adsorption of tetracycline.

Entry	Adsorbent	Adsorption capacity (mg/g)	adsorbent dosage (mg)	pollutant concentration (mg/L)	Reference
1	CTM@Fe_3_O_4_	215.31	50	60	[52]
2	Fe_3_O_4_@SiO_2_-Chitosan/graphene oxide nanocomposite	485.3	20	44.4	[98]
3	H_3_PO_4_-activated	308.33	30	100	[99]
4	CS-TCMA	20.85	30	50	[100]
5	**CS@TDI@EDTA@γ-AlO(OH)**	**555.5**	**3**	**30**	**This work**

## 6 Cost analysis of CS@TDI@EDTA@γ-AlO(OH)

Practical application of the CS@TDI@EDTA@γ-AlO(OH) for the adsorption of diazinon and tetracycline depends on its process cost which is estimated from the cost of reagents and the cost of energy in US$ per kg or US$ per m^3^ of pollutants, according to Eq. (15) [101].

(15) $$\text{Operating}\;\text{cost}=C_{\text{energy}}-\sum C_{\text{reagents}}$$

In this study, C_reagents_ is the cost of the reagents (US$) and C_energy_ is the energy consumption of furnace/oven (US$). The calculated operating cost for removal of diazinon and tetracycline under optimum operating conditions (adsorbent dose = 3 mg, volume = 50 mL, pH = 7 and 9, CT = 60, time = 120 min, T = 60 °C) was ∼15 US$ per m^3^. This value is indicative of the economic efficiency of the synthesized adsorbent for removal of diazinon and tetracycline.

## 7 Conclusions

In this study, CS@TDI@EDTA@γ-AlO(OH) composite was designed and prepared as a reusable and high efficiency adsorbent for the removal of diazinon and tetracycline from aqueous solutions. Successful synthesis of CS@TDI@EDTA@γ-AlO(OH) adsorbent was confirmed by performing different spectroscopic analyses. The obtained results showed that CS@TDI@EDTA@γ-AlO(OH) has a high affinity to adsorb diazinon and tetracycline. In addition, maximum adsorption capacity for diazinon and tetracycline reached 1425.5 and 555.5 mg/g, respectively. According to kinetic studies, the adsorption of diazinon and tetracycline on CS@TDI@EDTA@γ-AlO(OH) follows pseudo-second-order model. The suitability of adsorption data for diazinon and tetracycline was confirmed by using Langmuir isotherm model and Freundlich model. Thermodynamic data also revealed that the adsorption of diazinon and tetracycline on CS@TDI@EDTA@γ-AlO(OH) is exothermic in nature.

## Data availability statement

Data will be made available on request.

## CRediT authorship contribution statement

**Amir Adibzadeh:** Writing – review & editing, Writing – original draft, Software, Methodology, Formal analysis, Data curation. **Mohammad Reza Khodabakhshi:** Writing – review & editing, Writing – original draft, Supervision, Software, Project administration, Methodology. **Ali Maleki:** Writing – review & editing, Writing – original draft, Software, Methodology, Formal analysis, Data curation.

## Declaration of competing interest

The authors declare that they have no known competing financial interests or personal relationships that could have appeared to influence the work reported in this paper.
