# Supplementary material for: Preparation of novel and recyclable chitosan-alumina nanocomposite as superabsorbent to remove diazinon and tetracycline contaminants from aqueous solution
Source: Heliyon. 2023 Dec 9;10(1):e23139. doi: 10.1016/j.heliyon.2023.e23139 (PMC10761367; doi:10.1016/j.heliyon.2023.e23139)
Supplement: Multimedia component 1 [file mmc1.docx]

**Supporting information**

**Preparation of novel and recyclable chitosan-alumina nanocomposite as superabsorbent to remove diazinon and tetracycline contaminants from aqueous solution**

Amir Adibzadeh ^a^, Mohammad Reza Khodabakhshi ^b^*, Ali Maleki ^c^

^a^Health Research Center, Life Style Institute, Baqiyatallah University of Medical Sciences, Tehran, Iran

^b^Applied Biotechnology Research Center, Baqiyatallah University of Medical Sciences, Tehran, Iran ^c^ Catalysts and Organic Synthesis Research Laboratory, Department of Chemistry, Iran University of Science and Technology, Tehran, Iran

[*khodabakhshi@bmsu.ac.ir](mailto:*khodabakhshi@bmsu.ac.ir)

**Scheme 1.** structure of diazinon, tetracycline and CS@TDI@EDTA@γ-AlO(OH).


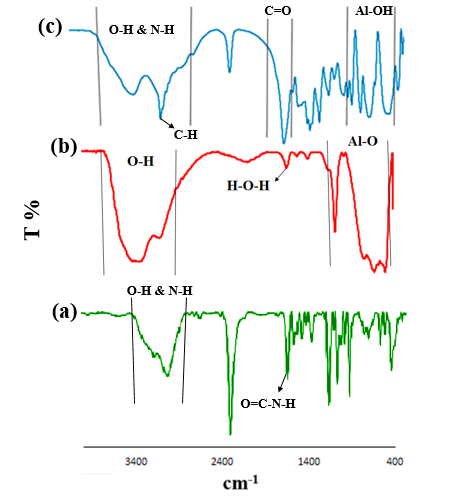


**Fig. S1.** FTIR spectra of the chitosan (**a**), BNPs (**b**) and CS@TDI@EDTA@γ-AlO(OH) (**c**).


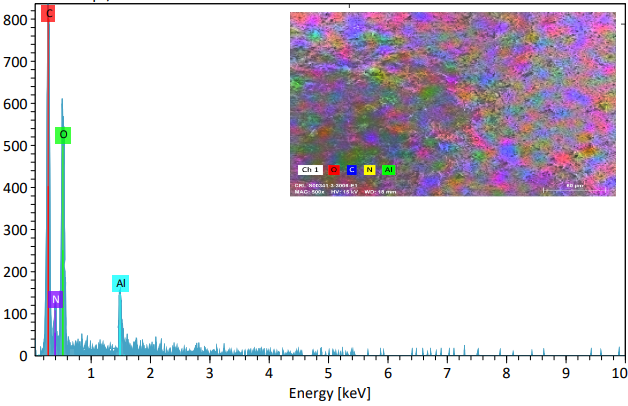


**Fig. S2.** EDX spectra of the Cs@TDI@EDTA@γ-AlO(OH).

**Fig. S3.** XRD patterns of the Cs@TDI@EDTA@γ-AlO(OH).

| 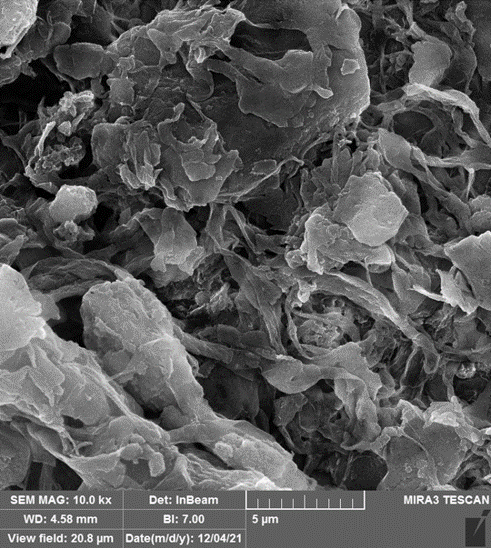  **(a)** | 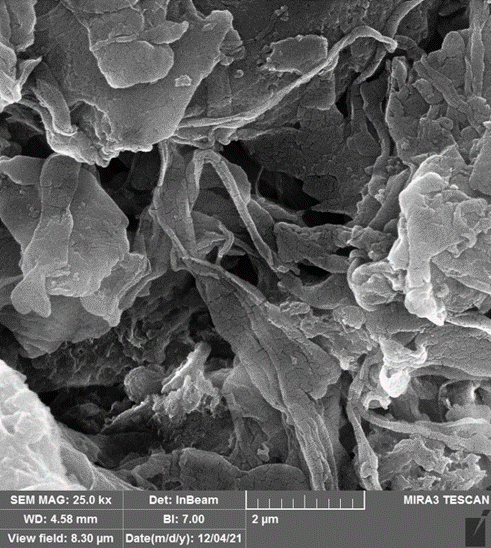  **(b)** |
| --- | --- |
| 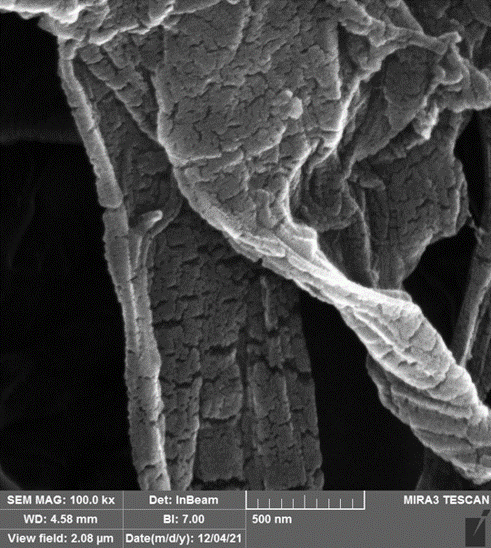  **(c)** | 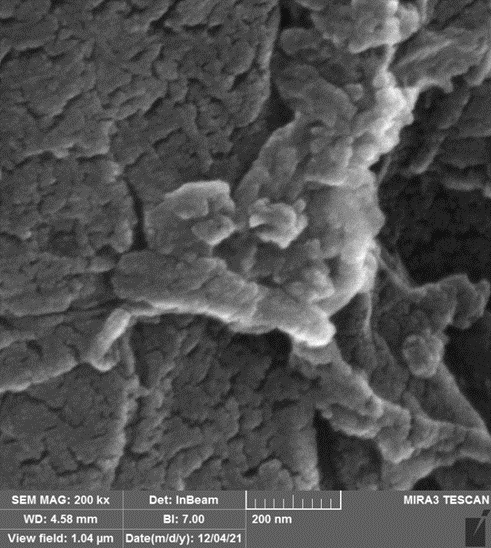  **(d)** |

**Fig. S4.** FESEM images of the Cs@TDI@EDTA@γ-AlO(OH).


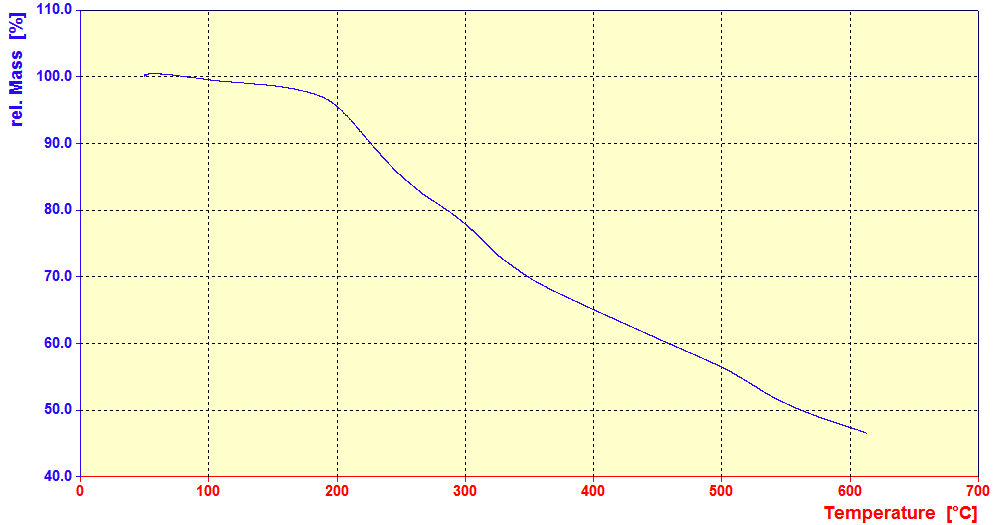


**Fig. S5.** TGA curve of the Cs@TDI@EDTA@γ-AlO(OH).

**Q (mg/g)**

**Fig. S6.** Effect of the of initial concentrations for the adsorption of diazinon (**a**) and tetracycline (**b**).

**Q (mg/g)**

**Q (mg/g)**

**(b)**

**Fig. S7.** Effect of the adsorbent dose for the adsorption of diazinon (**a**) and tetracycline (**b**).

**(b)**

**Fig. S8.** Effect of pH on the adsorption of diazinon (**a**) and tetracycline (**b**).

**Fig. S9.** Effect of temperature on the adsorption of diazinon (**a**) and tetracycline (**b**).

**(b)**

**Fig. S10.** Effect of the time on the adsorption of diazinon (**a**) and tetracycline (**b**).

**Fig. S11.** Pseudo-first-order model of diazinon (**a**) and tetracycline (**b**) on Cs@TDI@EDTA@γ-AlO(OH).

**Fig. S12.** Pseudo- second-order model of diazinon (**a**) and tetracycline (**b**) on Cs@TDI@EDTA@γ-AlO(OH).

**Fig. S13.** Langmuir isotherm model for diazinon (**a**) and tetracycline (**b**) on Cs@TDI@EDTA@γ-AlO(OH).

**Fig. S14.** Freundlich isotherm model for diazinon (**a**) and tetracycline (**b**) on Cs@TDI@EDTA@γ-AlO(OH).

**Fig. S15.** Temkin isotherm model for diazinon (**a**) and tetracycline (**b**) on Cs@TDI@EDTA@γ-AlO(OH).

**Fig. S16**. Adsorption-desorption cycles of diazinon and tetracycline on the Cs@TDI@EDTA@γ-AlO(OH).
